# A case report of spontaneous pectoral hematoma in a male with background antiplatelet therapy after severe COVID-19 infection

**DOI:** 10.1186/s12959-023-00539-7

**Published:** 2023-09-07

**Authors:** Hao Tang, Yan Yan

**Affiliations:** 1 Center for Coronary Artery Disease, Division of Cardiology, Beijing Anzhen Hospital, Capital Medical University, 2 Anzhen Road, Chaoyang District, 100029 Beijing, China; 2 National Clinical Research Center of Cardiovascular Diseases, Beijing, China; 3 Beijing Institute of Heart, Lung, and Blood Vessel Diseases, Beijing, China

**Keywords:** Spontaneous muscle hematoma, COVID-19, Male, Anticoagulation

## Abstract

**Background:**

Spontaneous muscle hematoma is a rare complication in hospitalized patients with COVID-19. We present a case of spontaneous pectoral hematoma occurring after COVID-19 infection and anticoagulation therapy.

**Case presentation:**

A 69-year-old male presented to the hospital with a two-week history of shortness of breath and a one-week history of high fever. Despite testing positive for COVID-19, the patient’s symptoms did not improve with two doses of ritonavir-boosted nirmatrelvir (Paxlovid). A chest CT scan revealed pulmonary infection and SpO_2_ tested between 80% and 85% at rest in local hospital. The patient transferred to our intensive care unit, then received multiple treatments, including high flow nasal oxygen (HFNO), antibiotics, methylprednisolone, IL-6 receptor antagonist monoclonal antibody (tocilizumab), and an increased D-Dimer level leaded to intermediate dose of anticoagulation therapy. However, on the 10th day of hospitalization, the patient developed a hematoma in the left pectoralis major muscle. This was accompanied by hemorrhagic shock, necessitating the administration of norepinephrine, fluid resuscitation, and a blood transfusion. Arterial embolization was performed to manage the bleeding, resulting in stabilization of the patient’s condition. Following discharge, the patient experienced an uneventful recovery over a period of six months.

**Conclusions:**

Severe COVID-19 patients undergoing routine therapeutic anticoagulation may experience fatal bleeding complications. The ideal dosage of anticoagulants for these patients remains uncertain, especially in the patient with a background of anticoagulation or dual antiplatelet therapy. We present a case of spontaneous muscle hematoma accompanied by hemorrhagic shock. The notable reduction in hemoglobin levels indicated significant bleeding, which was confirmed through contrast angiography and cured by arterial embolization. This case underscores the importance of additional research to determine the appropriate utilization of therapeutic anticoagulation in severe COVID-19 patients already undergoing antithrombotic therapy.

## Background

The COVID-19 Treatment Guidelines recommend that patients diagnosed with COVID-19, who are already receiving anticoagulant or antiplatelet therapies for underlying medical conditions, should continue their medication unless they experience significant bleeding or have other contraindications (AIII) [1]. Anticoagulation therapy, which is strongly recommended based on substantial evidence, is widely administered to hospitalized COVID-19 patients worldwide [2–9]. However, there is a lack of data regarding the efficacy, safety, and optimal dosage of anticoagulants in patients undergoing dual antiplatelet therapy (DAPT) or those with chronic use of oral anticoagulants as they were typically excluded from previous studies. Therefore, limited information exists about the effectiveness of anticoagulant treatment in these specific patient populations.

This case report describes a 69-year-old male patient who was admitted to the intensive care unit due to COVID-19 infection and required oxygen support. The patient had a medical history of hypertension, diabetes, hyperlipidemia and coronary artery disease, and had been receiving dual antiplatelet therapy within 6 months after stenting. We present the case of a male COVID-19 patient who received anti-inflammatory therapy and anticoagulation therapy prior to experiencing a spontaneous muscle hematoma and hemorrhagic shock. The clinical features, laboratory characteristics, and treatment of this patient are summarized. This case underscores the complexities and challenges involved in managing COVID-19 patients with comorbidities, the potential risks of bleeding complications, and the necessity of employing multidisciplinary approaches in the treatment of coagulation disorders.

## Case presentation

A 69-year-old male was admitted to the hospital on December 30, 2022, with complaints of “shortness of breath for 2 weeks and high fever for 1 week”. The patient had no identifiable triggers for the shortness of breath and mild fever that began 2 weeks prior to admission and did not seek medical attention at that time. Ten days before admission, the patient tested positive for COVID-19 antigen and experienced fluctuating body temperature ranging between 37.5 and 38.2 °C, which was periodically managed with antipyretic medication. Subsequently, the patient visited a local hospital where a chest CT scan revealed diffuse multiple patchy shadows in both lungs, indicating pulmonary infection. Ritonavir-boosted nirmatrelvir (Paxlovid) was administered twice daily; however, the patient continued to experience hypoxia, with a resting SpO_2_ level ranging from 80 to 85%. As a result, the patient was transferred to our hospital for further treatment. The patient’s medical history includes hypertension, diabetes, and hyperlipidemia, for which he takes irbesartan and hydrochlorothiazide tablets (150 mg/12.5 mg), metformin, and atorvastatin, respectively. Additionally, the patient underwent percutaneous coronary intervention (PCI) with stent placement 5 months prior to admission due to unstable angina pectoris and currently receives daily aspirin and ticagrelor. Furthermore, the patient’s history includes a prior atrial fibrillation ablation procedure. In subsequent follow-up phases after the ablation, a conspicuous absence of any atrial fibrillation recurrence was observed. This pivotal outcome led to the elimination of the patient’s protracted necessity for anticoagulant therapy.

**Fig. 1:**
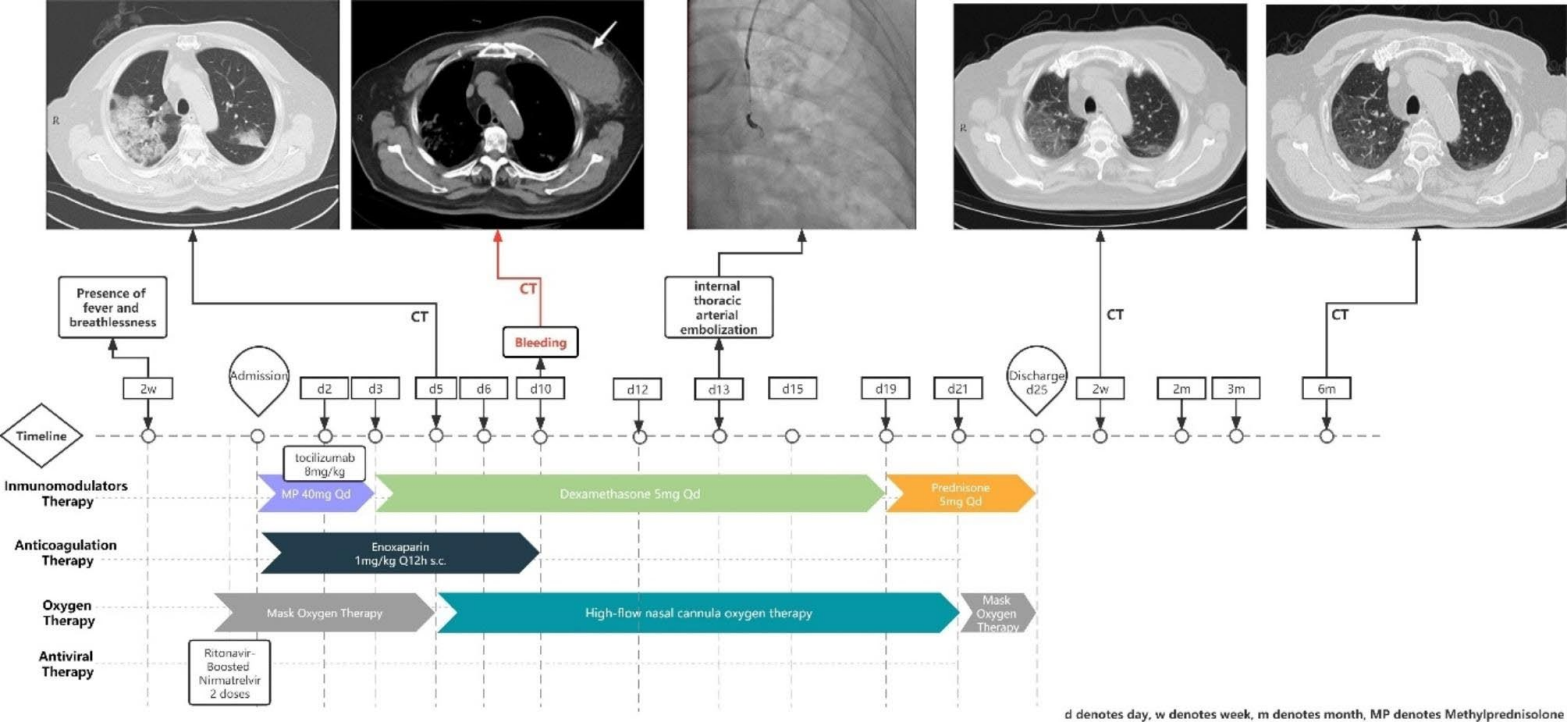
Scheme of medical treatment and intervention during hospitalization and follow-up

Upon admission, a physical examination revealed audible moist rales in both lungs. Blood tests indicated the following results: white blood cell count of 18.04 × 10^9^/L (normal range: 3.5 × 10^9^~9.5 × 10^9^/L), lymphocyte count of 0.48 × 10^9^/L (normal range: 0.8 × 10^9^~4.0 × 10^9^/L), hemoglobin level of 139 g/L (normal range: 120 ~ 160 g/L), C-reactive protein level of 198.45 mg/L (normal range: 0 ~ 8.2 mg/L), aspartate aminotransferase level of 42U/L (normal range: 8 ~ 40U/L), albumin level of 28.9 g/L (normal range: 40 ~ 55 g/L), and potassium level of 3.38mmol/L (normal range: 3.5 ~ 5.5 mmol/L). Blood gas analysis yielded the following results: pH of 7.483, pCO_2_ of 27.6 mmHg, and pO_2_ of 70.6 mmHg. Figure 1 depicts the radiology and coagulation tests performed during the patient’s hospitalization, while Table 1 presents the corresponding results. Due to the patient’s previous use of ritonavir-boosted nirmatrelvir, the dual antiplatelet therapy (DAPT) regimen was modified from aspirin with ticagrelor to aspirin with clopidogrel. The patient was placed in a prone position and received high-flow nasal oxygen (HFNO), along with medications including antibiotics, methylprednisolone, IL-6 receptor antagonist monoclonal antibody (tocilizumab), and an intermediate dose of enoxaparin.

**Table 1:** Key blood tests during hospitalization and follow-up

Variables	ER	In-hospital								Follow-up			
	Admission	D1	D3	D4	D6	D11	D15	D19	D22	2 weeks	2 months	3 months	6 months
Hb, g/l	137	139	135	147	140	76	97	110	122	136	147	153	155
SpO_2_, %	97	92.7	81.6	92.5	93.8	N/A	N/A	N/A	N/A	98.8	96.4	96.3	N/A
D-Dimer, ng/ml	6543	7074	3527	2487	4227	768	929	1809	1652	1078	220	168	74
FDP, µg/ml	N/A	55.8	21.8	14.6	28.2	5.3	8.4	13.6	12.3	8.78	1.16	1.16	N/A
FBG, g/l	4.82	5.6	3.6	2.9	2.2	1.2	2.1	2.3	2.7	3.8	3.1	3.39	N/A
APTT, s	25.8	25.5	28.2	30.3	28.5	20	22.4	24.8	26.7	28.7	32.5	32.2	N/A
PT, s	14.8	13.5	12.3	12.8	13	11.5	12.6	12.1	12.5	11.4	12.3	12	N/A
PLT, *10^9/l	231	248	251	250	212	208	134	171	188	259	200	217	214

D denotes day and N/A not available

On the 10th day of hospitalization, the patient reported left-sided chest pain and difficulty breathing. There was a significant increase in skin tension over the left chest, and a chest CT scan revealed a hematoma in the left pectoralis major muscle (Fig. 1). Following confirmation of spontaneous muscle hematoma complications, all antithrombotic therapies, including aspirin, clopidogrel, and enoxaparin, were discontinued. Conservative therapy involving the application of a bandage was initiated, and the patient’s hemoglobin level was monitored daily (Table 1). However, despite the use of norepinephrine, fluid resuscitation, and transfusion of 4 units of red blood cells, the patient’s hemoglobin level dropped by more than 5 g/L. Subsequently, the patient, who was initially diagnosed with hemorrhagic shock, underwent a critical and time-sensitive arterial angiography procedure aimed at identifying the precise origin of the hematoma. Despite thorough efforts, the bleeding vessel remained elusive within a limited timeframe. Given the noteworthy dilation of the internal thoracic artery, it was deemed a potential candidate for the source of bleeding. Consequently, a decision was made to proceed with an intervention known as internal thoracic arterial embolization. Subsequent soft tissue ultrasound examinations revealed ongoing absorption of the hematoma, and the patient’s condition stabilized after the arterial embolization procedure. On the third day following the procedure, the PRECISE-DAPT score was reassessed and found to be 42. Following a thorough evaluation of the patient’s ischemic and bleeding risk, it was determined that a modification in the duration of dual antiplatelet therapy was warranted. As a result, the decision was made to switch the patient to clopidogrel, prescribed at a daily dosage of 75 mg, effective upon discharge.

During the 6-month follow-up period, the patient underwent regular blood tests and CT scans. A computed tomography pulmonary angiogram was performed during a vital visit to evaluate for possible complications such as pulmonary embolism. Fortunately, the patient experienced a smooth recovery without any significant events.

## Discussion

The inflammatory cytokine storm triggered by COVID-19 can lead to endothelial damage and activation of the body’s coagulation system, resulting in a hypercoagulable state. Therefore, anticoagulation therapy has been regularly proposed to mitigate the risk of thrombotic events after COVID-19 [1, 2]. However, few randomized studies have evaluated the optimal dose and duration of routine anticoagulant in the critically ill patients with background of DAPT, despite its widespread utilization globally [7]. In contrast to intermediate dose of anticoagulant performed in more stable clinical conditions, there was no consciousness on optimal dose for sever patients except when there is a separate indication for anticoagulation [10, 11]. Apart from issues regarding the efficacy of the anticoagulation treatment, the safety profile of such interventions should be carefully considered. It is worth noting that the therapeutic dosage of anticoagulants was associated with increased risk of significant bleeding events compared with prophylactic anticoagulation among the abovementioned studies, corresponding to local preference for COVID-19 patients. Additionally, we analyzed the factors that might have contributed to the bleeding in this case: (1) The antiviral therapy involving ritonavir boosted nirmatrelvir has the potential to inhibit P450 enzymes, potentially impacting the metabolism of certain antiplatelet agents, such as ticagrelor. However, it seems improbable that this was the contributing factor in this specific case report. This assertion stems from the transient nature of drug-drug interactions, which typically endure for a span of 2–3 days post-antiviral therapy [12]. Significantly, the occurrence of bleeding in this case transpired on the 11th day subsequent to the completion of antiviral treatment. (2) The utilization of dexamethasone for anti-inflammatory treatment has the potential to suppress platelet function. This effect should be taken into account when evaluating the patient’s response to therapy and considering potential factors that could influence platelet-related outcomes [13]. (3) Furthermore, it is imperative to consider the patient’s positioning during the course of treatment. The patient was subjected to prolonged periods (12–16 h per day) in a prone position from the time of admission until the onset of the bleeding episode. This extended prone positioning can create mechanical shear stress between the chest’s soft tissues and the bed’s surface, which in turn could potentially lead to vascular injury in the chest area, as indicated by recent research [14]. Notably, the effects of prolonged and forceful coughing on vascular integrity have also been documented [15]. Given these factors, it is important to acknowledge the plausible influence of both prone positioning during ventilation and bouts of intense coughing as potential contributing factors to the observed bleeding event. In the broader context of employing anticoagulation therapy in COVID-19 patients, it is crucial to undertake a thorough assessment of both its effectiveness and safety. This evaluation should consider the unique clinical attributes of each patient as well as the potential for drug interactions. By tailoring the anticoagulation approach to the individual’s specific needs and medical history, we can strive to strike a balance between maximizing the therapeutic benefits and mitigating any associated risks.

Spontaneous muscle hematomas, including pectoralis major hematoma, have been observed as major bleeding complications in patients undergoing therapeutic anticoagulation. Interestingly, previous studies have reported that pectoralis major hematoma occurred exclusively in females, [16, 17]. but in our case report, we present a male patient with a spontaneous pectoralis major hematoma. In The Therapeutic Anticoagulation versus Standard Care as a Rapid Response to the COVID-19 Pandemic (RAPID) trial, [3] which focused on moderately ill patients with COVID-19 and elevated D-dimer levels admitted to hospital wards, therapeutic heparin did not show a significant reduction in the primary outcome, but it did decrease the odds of death at 28 days. However, it is important to note that the trial’s key exclusion criteria, which included indications for therapeutic anticoagulation and dual antiplatelet therapy, excluded patients at a higher risk of bleeding. Therefore, the risk of major bleeding in this trial appeared to be low. In the Intermediate vs. Standard-Dose Prophylactic Anticoagulation in Critically-ill Patients With COVID-19: An Open Label Randomized Controlled Trial (INSPIRATION) study, [3] comparing intermediate-dose enoxaparin with prophylactic-dose anticoagulation, the use of intermediate-dose enoxaparin was associated with a twofold increase in major bleeding and clinically relevant nonmajor bleeding, but it did not statistically reduce the occurrence of thrombotic events. Currently, there is no consensus on the treatment of spontaneous bleeding in COVID-19 patients, and clinical decision-making should be based on personalized analysis considering the patient’s specific circumstances to determine the need for interventional treatment. Previous studies have shown the effectiveness and safety of percutaneous arterial embolization in spontaneous hematomas in 9 patients with COVID-19 infection after anticoagulation therapy [18]. In our reported case, after confirming the spontaneous pectoralis major hematoma and unstable vital signs in the patient, immediate selective arterial angiography was performed to identify the responsible vessel, followed by arterial embolization. It is important to exercise caution regarding bleeding risk in COVID-19 patients with a history of antithrombotic therapy, although arterial embolization proved effective in this particular case.

## Conclusion

The present case reinforces the concept that routine therapeutic anticoagulation to COVID-19 patients with antithrombotic therapy background is subject to investigation. Nevertheless, if intermediate dose anticoagulation is considered in cases at higher risk of early thrombotic events, the safety of this strategy, assuming the use of multi drugs and respiratory support, is reassuring. Whether anticoagulant should be preferred with prophylactic dose in this kind of patients for a favorable efficacy-to-safety ratio serves confirmation in further studies.

## Data Availability

Not applicable.
